# Statistical learning methods as a preprocessing step for survival analysis: evaluation of concept using lung cancer data

**DOI:** 10.1186/1475-925X-10-97

**Published:** 2011-11-08

**Authors:** Madhusmita Behera, Erin E Fowler, Taofeek K Owonikoko, Walker H Land, William Mayfield, Zhengjia Chen, Fadlo R Khuri, Suresh S Ramalingam, John J Heine

**Affiliations:** 1 Department of Hematology and Medical Oncology, Emory University, Winship Cancer Institute, 1365 Clifton Road NE, Rm C-3090, Atlanta, GA 30322, USA; 2 Department of Cancer Epidemiology, H. Lee Moffitt Cancer Center & Research Institute, 12902 Magnolia Drive, MRC-CANCONT, Tampa, FL 33612, USA; 3 Thomas J. Watson School of Engineering, Binghamton University, State University of New York, PO Box 6000, Binghamton, NY 13902-6000, USA; 4 WellStar Kennestone Hospital, Marietta, GA 30060, USA; 5 Biostatistics & Bioinformatics Shared Resource at Wnship Cancer Institute, Department of Biostatistics & Bioinformatics, Rollins School of Public Health, Atlanta, GA, USA

## Abstract

**Background:**

Statistical learning (SL) techniques can address non-linear relationships and small datasets but do not provide an output that has an epidemiologic interpretation.

**Methods:**

A small set of clinical variables (CVs) for stage-1 non-small cell lung cancer patients was used to evaluate an approach for using SL methods as a preprocessing step for survival analysis. A stochastic method of training a probabilistic neural network (PNN) was used with differential evolution (DE) optimization. Survival scores were derived stochastically by combining CVs with the PNN. Patients (n = 151) were dichotomized into favorable (n = 92) and unfavorable (n = 59) survival outcome groups. These PNN derived scores were used with logistic regression (LR) modeling to predict favorable survival outcome and were integrated into the survival analysis (i.e. Kaplan-Meier analysis and Cox regression). The hybrid modeling was compared with the respective modeling using raw CVs. The area under the receiver operating characteristic curve (Az) was used to compare model predictive capability. Odds ratios (ORs) and hazard ratios (HRs) were used to compare disease associations with 95% confidence intervals (CIs).

**Results:**

The LR model with the best predictive capability gave Az = 0.703. While controlling for gender and tumor grade, the OR = 0.63 (CI: 0.43, 0.91) per standard deviation (SD) increase in age indicates increasing age confers unfavorable outcome. The hybrid LR model gave Az = 0.778 by combining age and tumor grade with the PNN and controlling for gender. The PNN score and age translate inversely with respect to risk. The OR = 0.27 (CI: 0.14, 0.53) per SD increase in PNN score indicates those patients with decreased score confer unfavorable outcome. The tumor grade adjusted hazard for patients above the median age compared with those below the median was HR = 1.78 (CI: 1.06, 3.02), whereas the hazard for those patients below the median PNN score compared to those above the median was HR = 4.0 (CI: 2.13, 7.14).

**Conclusion:**

We have provided preliminary evidence showing that the SL preprocessing may provide benefits in comparison with accepted approaches. The work will require further evaluation with varying datasets to confirm these findings.

## Background

Statistical learning (SL) techniques with kernel mappings can provide benefits when addressing complicated decision problems [1-3]. These techniques are capable of capturing non-linear input-output characteristics, operating on small datasets with feature correlation, and do not require modeling or distribution assumptions. These attributes are not derived without tradeoffs. These methods do not provide an output that has a useful epidemiologic interpretation and their training often requires specialized techniques. In contrast, logistic regression (LR) modeling, Kaplan-Meier analysis, and Cox regression provide important epidemiologic interpretations and are used extensively due to their availability. This report is an advancement of our earlier simulation work [4] in adapting SL methods for epidemiologic application (see Appendix).

Lung cancer is the leading cause of cancer-related mortality in the world with more than a million deaths each year [5]. Lung cancer is often diagnosed at an advanced stage since early detection has been elusive [6]. Recent evidence indicates that lung cancer mortality can be reduced when screening high-risk patients with a low-dose computerized tomography (CT) scan [7]. Before this promising approach is incorporated into general practice, several important outstanding clinical issues have to be addressed [6,7]. For patients with early stage lung cancer, local therapy with surgical resection is associated with the best survival outcomes. This is limited to those with non-small cell lung cancer (NSCLC), which accounts for approximately 85% of all cases of lung cancer in the United States. Despite optimal surgical resection, recurrence of disease is noted in 30-75 percent of the patients based on the initial stage. Development of prognostic models for predicting survival outcomes for patients with NSCLC after resection will have important healthcare implications.

To adapt an SL methodology for epidemiologic application, a problem in NSCLC survival prognosis was analyzed for stage-1 patients using a relatively small set of variables collected routinely for patients of this kind, similar to those investigated previously [8]. A probabilistic neural network (PNN) [9] was combined with LR modeling and survival analyses (i.e. Kaplan-Meier analysis and Cox regression) to demonstrate proof of concept. This hybrid approach combines the strengths of the SL methodology with these important epidemiologic techniques. The PNN is a statistically inspired neural network [9] that uses a kernel mapping [10,11] to estimate the underlying probabilities. For the LR modeling comparisons, the NSCLC dataset was dichotomized into two groups comprised of patients with favorable or unfavorable survival outcomes. Raw clinical variables and a new patient score variable formed with the modified PPN were considered as prognostic factors. Additionally, the PPN output was used as the study variable and compared with age using survival analysis. There are weight parameters within the PNN that must be estimated properly. Differential evolution (DE) was used for this optimization problem [12]. Stochastic methods were developed to provide feedback to the DE optimization and to derive the patient PNN scores. We also evaluated this new system with the simulated datasets and methods described previously [4], as discussed in the Appendix.

## Methods

### Dataset

The dataset was comprised of data from 151 NSCLC patients that underwent surgical resection from 2002 - 2006. All data were selected retrospectively and consecutively. Stage-1 patients that had complete case ascertainment for the variables under consideration were selected. Ninety-two (n_1_) of these patients were alive at last contact (censored), and 59 (n_2_) patients died (incident) during the course of the contact interval. The clinical variables abstracted from the patient files included age (i.e. age of the patient at the time of procedure), gender (binary), history of smoking (binary), histology sub-type (four categories), and tumor grade. Past or current smokers were categorized as smokers (yes), otherwise patients were characterized as non-smokers (no). The four histological sub-types were: adenocarcinoma (AC), squamous cell carcinoma (SCC), large cell carcinoma (LCC), and adenosquamous carcinoma (ASC). Tumor grade is a 1-3 integer scale describing cancer cell differentiation (a measure of abnormality) derived from pathology reports. This data was collected under an approved protocol by the Western Institutional Review Board.

### Modeling Techniques

#### Favorable Outcome and Survival Analysis

The non-interaction LR model [13] was used to predict favorable and unfavorable survival outcome by dichotomizing the population into two groups. The 92 censored patients were designated as the favorable survival outcome group defined as group-1 (i.e. the censored group). Fifty-nine patients were designated as the unfavorable survival outcome group defined as group-2 (i.e. the incident group). Other methods of dichotomizing the population were considered but discarded as discussed in the Results Section. Overall survival (OS) time was measured as the distance between the date of procedure and the date of death for a given patient when applicable. Censor time was measured as the distance between the date of the procedure and the date that a given patient was censored, when applicable. The LR model was referenced to predict the probability of a favorable outcome. Age was treated as a continuous variable with integer accuracy, and grade was considered as a three state continuous integer variable (grades 1-3). Histology (four-state) and gender (two-state) were treated as categorical variables. Age and grade were combined to form a continuous patient score (or z) using a variation of the PNN. The reasons for this follow from the LR modeling (non-hybrid) findings and that they were treated as continuous variables, whereas the remaining variables were categorical or binary and not strictly amendable to probability density estimations. Kaplan-Meier product-limit estimators and Cox regression were used for the survival probability curve analyses. In this analysis, two groups were formed by choosing the median age and median PNN score as the separation points. The other relevant variables were introduced with both age and PNN score to evaluate their influence on the respective survival probability curves.

For the LR modeling comparisons, odds ratios (ORs) were used to assess measurement association. For age and PNN score (i.e. the continuous variables), the LR model coefficients were re-scaled to provide ORs per standard deviation (SD) change for each variable. The ORs for grade were cited in per unit increase. The area under the receiver operating characteristic curve (Az) was used to measure the predictive capability for a given model. The Az was estimated with three methods. First, to assess the SL training and patient scores, the definition of Az was applied [14] using the respective distributions. Secondly, the Az quantities for the LR models were generated within the SAS (SAS Institute, NC) software package using the output of the LR model (same interpretation as provided by the fist method). For the Kaplan-Meier analysis, chi-square Wilcoxon (more sensitive to shorter term survival differences) and log-rank (more sensitive to longer term survival differences) tests were used for differences in stratification. Hazard ratios (HRs) were estimated with Cox Regression. Thirdly, Az was also derived from Cox regression and is a measure of the agreement between the model and actual time-to-event outcome [15]. For the ORs and HRs 95% confidence intervals (CI) were provided. The survival analysis was also performed with SAS.

#### Probabilistic Neural Network and Kernel Methods

We implemented a variation of the PNN using a Gaussian kernel, although there are many kernels meeting the established criteria [16]. Paralleling our earlier work [17], the distance metric for a d dimensional input vector (i.e. the relevant clinical variables) is given by

(1) $${\text{D}}_{\text{i}}\left(w\right)=\overset{\text{d}}{\underset{\text{j}=1}{\sum }}\frac{{\left({\text{w}}_{\text{j}}-{\text{w}}_{\text{ij}}\right)}^{2}}{\sigma_{\text{j}}^{2}},$$

where i is the patient index, w_ij _is the j^th ^component of the i^th ^sample's input vector, and w_j _is the j^th ^component of a prospective test sample's input vector **w**. The sigma-weights, σ_j _, were estimated with DE optimization. Specifically, d = 2, with w_i1 _= age, and w_i2 _= grade for the i^th ^patient. The probability density estimation [10,11] for **w **with n training samples is expressed as

(2) $$\text{g}\left(w\right)=\frac{1}{\text{n}}\overset{\text{n}}{\underset{\text{i}=1}{\sum }}\exp\left[-{\text{D}}_{\text{i}}\left(w\right)\right]=\frac{1}{\text{n}}\overset{\text{n}}{\underset{\text{i}=1}{\sum }}\text{k}\left(w,w_{\text{i}}\right).$$

Normalization factors are discussed below. The PNN was constructed with the above formulism for each group. For group-1, the density for **w **is given by

(3) $${\text{g}}_{1}\left(w\right)=\frac{1}{{\text{n}}_{1}}\overset{{\text{n}}_{1}}{\underset{\text{i}=1}{\sum }}\text{k}\left(w,w_{\text{i}}\right).$$

For a given **w**, the sum on **w**_i _is taken over group-1 samples only with n = n_1_. The g_2_(**w**) density was estimated the same way by restricting the sum on **w**_i _to the group-2 samples with n = n_2_. In both the g_1 _and g_2 _estimations, **w **included samples from both groups. Equation (3) also represents a function mapping of the vectors **w **and **w**_i _, where each element of the summation represents the inner product of the mapped vectors [3], rendering a nonlinear problem tractable with the proper choice of kernel. Assuming prior probabilities and misclassification costs are equal, the PNN classifier [9] is expressed as

(4) $$\frac{{\text{g}}_{1}\left(w\right)}{{\text{g}}_{2}\left(w\right)}>\text{c},$$

where c is a constant. For classification when this condition is met, **w **belongs to group-1. Because we were interested in developing a score for each patient (not classification), we formed this score with the above expression

(5) $$\text{patient}-\text{score}=\frac{{\text{g}}_{1}\left(w\right)}{{\text{g}}_{2}\left(w\right)}.$$

The multivariate normalization factors were not important because both g_1 _and g_2 _contained the same sigma-weights. These scores were used with LR modeling and the survival analysis. Because the above expression is always positive and can be large, we used z = ln (patient - score) in the analyses as the PNN derived patient score and performed a range compression technique to reduce statistical outlier interference in the LR modeling.

#### Probabilistic Neural Network Training and Operation

**Figure 1:**
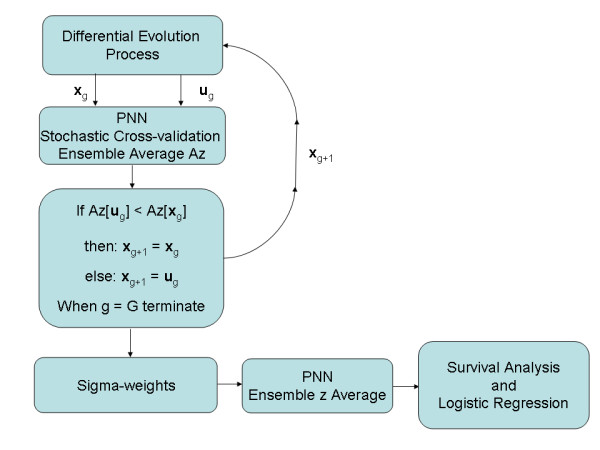
**Modified probabilistic neural network stochastic training and z generation flow diagram**. This schema shows the modified probabilistic neural network (PNN) training flow for the differential evolution (DE) sigma-weight vector construction, competition, and feedback from the g to the g+1 populations. The sigma-weight vectors **x**_g _and **u**_g _compete for the next generation. The receiver operating characteristic curve area (Az) from the stochastic cross-validation is derived with ensemble averaging to reduce the chance of passing outliers back to the vector competition. When g = G, the evolution stops and the sigma-weights are used in the PNN to generate z for each patient stochastically with ensemble averaging. The z quantities are then passed to the survival and logistic regression analyses.

The sigma-weights for the kernel in the PNN must be estimated properly. A stochastic cross-validation technique was developed in combination with DE to estimate these weights. DE is also a stochastic global optimization strategy that is self-organizing via feedback. We developed the algorithm described by the founders of DE [12] and used their notation in this description. Important points underlying DE were discussed in our previous work [17] and are briefly discussed here. We used a uniform crossover Cr = 0.9 and scale factor F = 0.2. The zero-generation feature vector population (i.e. NP = 40 vectors) was initialized with uniformly distributed random variables with components constrained to this range [0.01, 1.5]. For a given generation, the DE process constructs a mutant vector (or **v**_g_) by stochastic perturbation from the current population of **x**, where g is the generation index. From this, a candidate vector (or **u**_g_) is constructed that competes with a given current generation vector, **x**_g _, selected at random in such a way that it was not involved with the **v**_g _(or **u**_g_) construction. Possible solutions (**x**_g _and **u**_g_) compete against each other using feedback from the optimization problem. The winner moves to the next generation of **x **(i.e. g+1). For a given generation there are NP competitions. In our DE application, Az was the feedback measure using the two patient-score distributions derived from Eq. (5). The feedback to the DE was formed by ensemble averaging derived with bootstrap sampling [18]. For one DE generation, N_t _bootstrap populations were generated. To form a given bootstrap population, n_2 _samples were selected randomly from group-1 and from group-2 with replacement. We keyed on n_2 _as not to bias the sampling to the larger population. One sample from each class was selected randomly and used as **w **in Eq. (5) to generate the respective patient-score quantities. The remaining samples were used to build the respective **w**_i _populations in Eq. (5). We refer to this process as a leave two-out stochastic cross-validation technique. When N_t _= 1, the process is similar to the conventional leave two-out approach using different realizations of the population. This process then was repeated N_t _(i.e. training) times and the average Az was used as feedback for one DE generation. The process was terminated after G generations. The weights that provided the largest Az were carried over to the analysis and used to generate z for each patient using stochastic methods and ensemble averaging. For a given **w**, a bootstrap population was generated from the **w**_i _population and the respective z was generated for all n_1 _and n_2 _patients. Each patient's z was derived from ensemble averaging by repeating this process for N_sc _times. The training process and final score generation flow are shown in the Figure 1 schema. As provided in the Appendix, we assessed the system described above with both simulated data using the same methods described previously [4] and with holdout cross-validation techniques to show internal validity. The software for the PNN and DE applications was developed by the authors using the IDL (ITT Visual Information Solutions, Boulder CL) programming language.

## Results

### Favorable Outcomes

The patient characteristics are summarized in Table 1. To assess inter-group differences in these clinical variables, a t-test was used for continuous variable comparisons, and the binomial proportional test (with the normal approximation) was used for the categorical or binary variable comparisons, where applicable. As shown in the right most column of Table 1, the censored group patients are younger, more likely female, tend to have grade 1 disease, and the smoking status is similar. The censored group is more likely to have AC in comparison with the incident group, whereas the other histology-type and other grade differences are less clear in the summary format. The standard LR modeling (i.e. raw clinical variables or accepted approach) findings are shown in Table 2 (top). A one SD increase in age (SD = 8.68 years) indicates the respective patient is 0.60 times more likely to be in group-1 (or 1.66 times more likely to be in group-2 and not survive), which was significant. The OR for the tumor grade adjusted model shows a trend. A unit increase in grade indicates a given patient is about 1.5 times more likely to be in group-2, but this association was not significant. When introducing gender with age and grade, the gender association OR = 0.38 was significant indicating females are 2.6 times more likely have a favorable outcome. The adjustments for grade and gender had a minor influence on the age ORs. However, adding these variables increased the model's predictive capability: Az = 0.636 (age alone), Az = 0.657 for age and grade, and Az = 0.703 for age, grade, and gender. The standard error (SE) in Az was estimated as SE = 0.03. Adding histological-type and smoking status with gender and grade produced marginal influences on the relationships (not shown). In summary, younger age, lower-grade, and gender (female) confer a favorable survival outcome. The weak association for grade with survival could be due to limited samples, the association is truly marginal, or the relationship is complicated and cannot be captured with the LR model. To assess these possibilities, grade was combined with age using a variation of the PNN classifier.

**Table 1:** Patient characteristics.

Characteristic	Incident n	Incident mean/SD or %	Censored n	Censored mean/SD or %	p-value
Age	59	69.58/7.85	92	65.42/8.84	0.0038*
					
Grade	59	2.22/0.62	92	2.10/0.68	0.2651*
One	6	10.17%	17	18.48%	0.1656
Two	34	57.63%	49	53.26%	0.5988
Three	19	32.20%	26	28.26%	0.6053
					
Gender					
Male	38	64.41%	34	36.96%	0.0010
Female	21	35.59%	58	63.04%	0.0010
					
Histology subtype					
Adenocarcinoma	29	49.15%	58	63.04%	0.0919
Squamous	25	42.37%	20	21.74%	0.1510
Large Cell	3	5.08%	11	11.96%	0.1555
Adenosquamous	2	3.39%	3	3.26%	0.9655
					
Smoking status					
Non-Smoker	12	20.34%	19	20.65%	0.9629
Smoker	47	79.66%	73	79.35%	0.9629

The patient characteristics are summarized in this table. The incident column refers to group-2 patients (unfavorable survival outcome) and the censored column refers to group-1 patients (favorable survival outcome). The mean and standard deviation (SD) are provided for the continuous variables and percentages (%) are provided for the other variables by group. The number of patients (n) for each variable is given for each group. The incident and censored group characteristics were compared with either the t-test (*) or binomial proportional test. The relevant p-values are provided in the last column (right).

**Table 2:** Odds Ratios.

Model	SD	Age OR	Az	Covariate	Unit	Covariate OR
Accepted						
Age	8.681	0.60 (0.42, 0.86)	0.636			
						
Grade adjusted	8.681	0.58 (0.40, 0.83)	0.657	Grade	1	0.68 (0.40, 1.15)
						
Grade and Gender adjusted	8.681	0.63 (0.43, 0.91)	0.703	Grade	1	0.73 (0.42, 1.25)
				Gender	Male vs Female	0.38 (0.19, 0.78)
						

Model	SD	ln(z) OR	Az	Covariate	Unit	Covariate OR

Hybrid						
z (Age and Grade)	1.695	4.15 (2.15, 8.01)	0.763			
						
Gender adjusted	1.695	3.67 (1.88, 7.16)	0.778	Gender	Male vs. Female	0.50 (0.24, 1.05)

The odds ratios (ORs) and 95% confidence intervals (CIs) are provided parenthetically for the variables used in the logistic regression modeling. The ORs for the continuous variables (age and z) are cited per standard deviation (SD) increase in the respective variable or as a unit increase (grade) while controlling for the other variables (covariates) when applicable. The z variable includes grade and age simultaneously. The ORs for the other covariates are listed in the column to the right. The area under the receiver operating characteristic curve (Az) is also provided for each model.

The DE training for the modified PNN resulted in two sigma-weights with σ_1 _= 0.013610961 and σ_2 _= 0.35805283 for age and grade, respectively. Using N_t _= 1 produced training Az values between 0.700-0.830. Choosing N_t _= 5 gave consistent findings and was used in the analysis. The stochastic cross-validation performance coinciding with these weights gave Az = 0.710 with SE = 0.03 after three generations (G = 3), which is in agreement with the Az derived from holdout cross-validation analysis (see Appendix). We used these parameters to generate z for each patient with N_sc _= 20. Processing age and grade separately through the PNN gave Az = 0.656 for age and Az = 0.538 for grade, which are statistically similar to the Az values when assessing these variables individually without the PNN processing. The continuous hybrid LR findings are shown in the bottom of Table 2. The combined effect shows that for a SD increase in z (SD = 1.69), the respective patient is about 4.15 times more likely to experience a favorable survival outcome (or incident group member is 0.24 more likely to experience a favorable outcome) with Az = 0.763, which was significantly larger (p = 0.0062) than that provided by the respective age and grade LR model. Due to the way the PNN was defined, increasing z was protective, whereas increasing in age was not. Adjusting for gender increased the predictive capability of the model with Az = 0.778 (SE = 0.03), although the gender OR lost significance. Gender also reduced the association for z with OR = 3.67 per standard deviation increase, which was a stronger association than provided by age in the corresponding model. The Az derived from the hybrid model (z and gender) was significantly greater than that of the corresponding LR model with age, grade, and gender (p = 0.0173). As above, including histology-type or smoking status with z had a marginal influence on the relationships (not shown).

**Figure 2:**
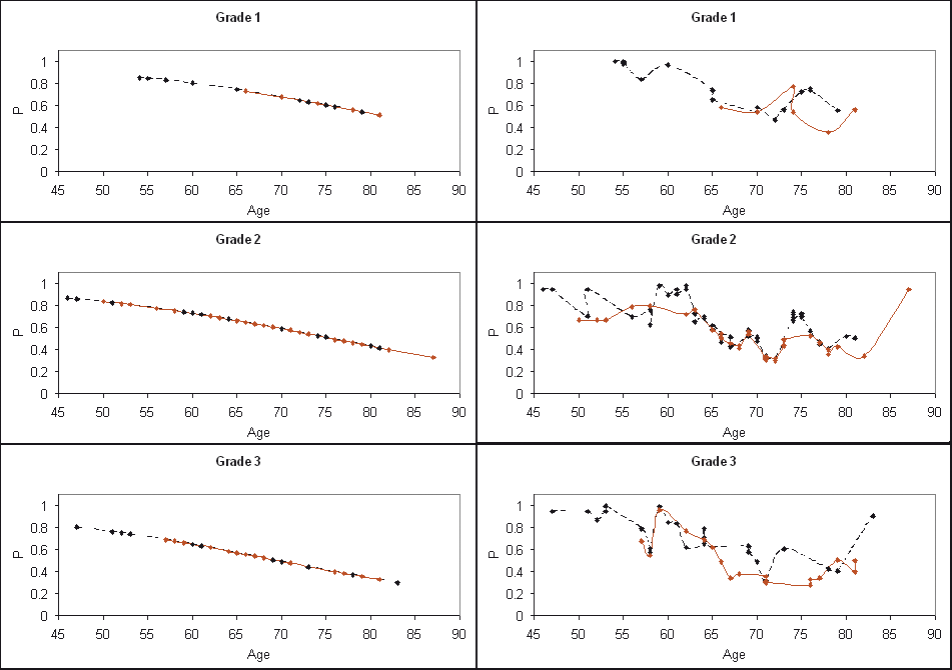
**Logistic regression model output for each tumor grade**. The plots on the left show the logistic regression model probabilities (P) using the age and grade variables as the model inputs for each tumor grade. The plots on the right show the respective hybrid logistic regression model probabilities (P) using the variable z (i.e. age and grade combined with the probabilistic neural network) as the model input. Because there are overlapping points (patients with the same grade and age), some points are not distinguishable. The censored group (black) is compared with the incident group (red). The curves were fitted with a cubic spline.

To evaluate the effect of the kernel mapping on age and grade, the LR model outputs for the two models were plotted as a function of grade and age. The left side of Figure 2 shows the grade plots for the LR (accepted approach with age and grade) model. The respective grade plots for the hybrid LR model using z (age and grade combined) are shown on the right side of Figure 2. In these plots black was used to denote censored group samples and red to denote incident group samples. The grade 1 plots for both models exhibit similar behavior for the lower ages and show that patients 65 years of age and younger are more likely to be in censored group. The hybrid model separates some older grade 1 patients in contrast with the accepted LR model. A comparison of the grade 2 plots shows that the hybrid model provides separation for the younger, middle age, and some upper age patients, whereas the respective accepted LR model produces confusion between the groups. In the grade 3 plots, both models provide separation for lower age patients, whereas the hybrid model shows group separation in the middle-age range as well. Because z is a composite variable and difficult to interpret, the associations between age, grade, z, and group status shown in Figure 2 are also summarized in Table 3. This provides the average values for age and the z variables separated by grade and group.

**Table 3:** Age and z relationships.

Censored group	Grade 1	Grade 2	Grade 3	All
n	17	49	26	92

Age (mean)	66.41	66.27	63.19	65.42
z (mean)	2.11	3.91	3.12	3.36

Incident group	Grade 1	Grade 2	Grade 3	All

n	6	34	19	59

Age (mean)	73.83	68.88	69.47	69.58
z (mean)	0.26	1.37	-0.07	0.8

This table gives the mean values for age and z as a function of tumor grade and censored/incident group status. The number (n) of patients in each category is also provided.

We used the OS and censor times to form two groups because of the separation between the respective distribution means. The favorable group had a mean censor time of 3.97 years (i.e. mean known OS time, which is a low-side limit assuming these patients did not expire the day after study-contact), whereas the incident group had a mean OS time of 2.20 years (data not shown). The minimum censor time (2.35 years) is greater than the mean OS time for the incident group indicating validity of the dichotomization method. Other methods of dichotomizing the population were considered, such as choosing a cutoff-point at given OS time but this technique added ambiguity with those censored on the left-side of the cut-point and left few samples on the right-side of the cut-point when considering four or five year OS times as the demarcation.

### Survival Analysis

**Figure 3:**
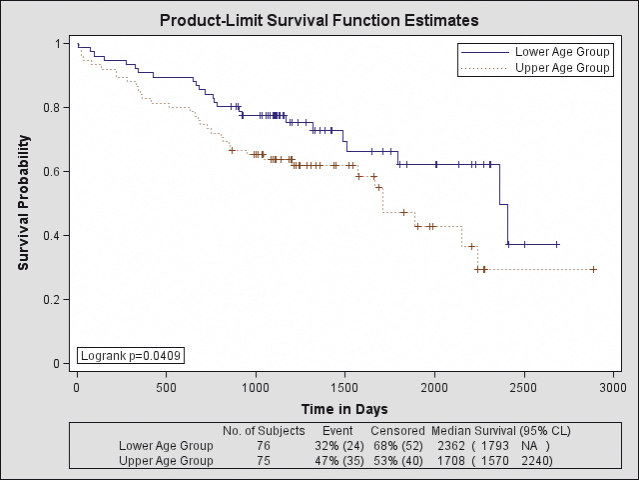
**Survival probability curves for age**. The upper and lower-age groups were formed by dichotomizing the total collection of patients at their median age. The lower-age curve (upper blue curve) exhibits better survival characteristics than the upper-age group (bottom brown curve).

**Figure 4:**
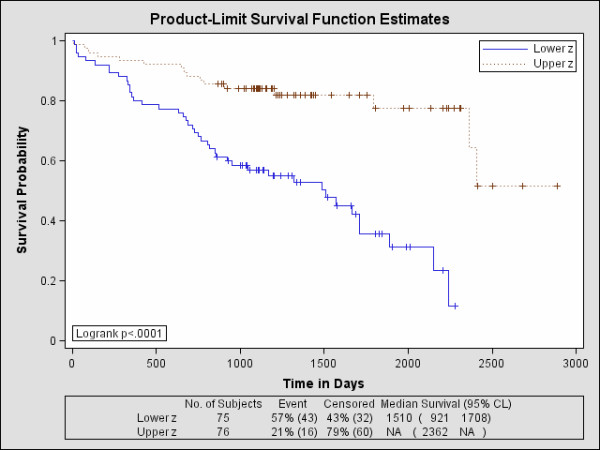
**Survival probability curves for z**. The upper and lower-z groups were formed by dichotomizing the total collection of patients at their median z value. The upper-z group (upper brown curve) exhibits better survival characteristics than the lower-z group (bottom blue curve). These findings incorporate tumor-grade with age via the probabilistic neural network combination.

The Kaplan-Meier survival probability curves for age are shown in Figure 3. The related findings are provided in Table 4. The hazard for age was HR = 1.72 indicating that upper-age group membership is significantly more hazardous than lower-age group membership. Roughly, 37% of the lower-age group survived past 7 years, whereas about 29% of the upper-age group survived past this time. The longer-term survival is significantly different between the two age groups (p < 0.050). Including grade induced a greater hazard with HR = 1.78, but the change in the survival curves when controlling for grade was not significant in either the short term (p = 0.074) or the longer-term (p = 0.091). The addition of gender caused a significant change in the survival curves compared with age alone for both the short term (p < 0.002) and long-term survival (p < 0.005) but the HR = 1.64 lost significance. The grade and gender adjusted hazard for age was HR = 1.68 (also lost significance). The statistical test findings for age and gender are provided in Table 5 (top rows). The survival probability curves for z are shown in Figure 4 and the HRs are provided in Table 4. There is a significant survival difference between these upper and lower-z groups both in the short term (p < 0.0001) and long term (p < 0.0001) with HR = 0.25 indicating those in the upper-z group are at a significantly reduced hazard compared with those in the lower z group (i.e. the hazard for those in the lower-z membership was HR = 4.0). About 52% of the upper-z group survived past 7 years, whereas as about 11% of the lower-z group survived past this time. The addition of gender also produced a significant change in both short term (p = 0.0146) and the longer term (p = 0.0319) with HR = 0.28 (HR = 3.57 for lower-z membership). The associated statistical comparisons for z and gender are provided in Table 5 (bottom two rows). As shown in Table 4, the hybrid Cox model (i.e. using z) showed greater concordance (Az = 0.691) with the outcome than that of the Cox model (accepted approach) using age and grade (Az = 0.606), but the difference in Az was a trend (p = 0.056). Likewise, the Az comparison between the hybrid Cox model using z and gender (Az = 0.738) with the Cox model using age, grade, and gender (Az = 0.677) showed a similar trend (p = 0.0747). The favorable prognostic values for both age and gender are in agreement with other studies [8,19].

**Table 4:** Hazard relationships for dichotomous age and z.

Model	Age Hazard Ratio	Az
Accepted		
Dichotomous Age	1.72 (1.02, 2.90)	0.5792
		
Grade adjusted	1.78 (1.06, 3.02)	0.606
		
Gender adjusted	1.64 (0.96, 2.78)	0.669
		
Grade Gender adjusted	1.68 (0.99, 2.85)	0.677
		

Model	z Hazard Ratio	Az

Hybrid		
Dichotomous z	0.25 (0.14, 0.47)	0.691
		
Gender adjusted	0.28 (0.15, 0.53)	0.738

For the age and z variables, two groups were formed using the respective distribution median as the cut-point and compared. The hazard ratios (HRs) are provided parenthetically with 95% confidence intervals. The area under the receiver operating characteristic curves (Azs) derived from Cox regression models are also provided. Because age and z translate inversely with respect to hazard, increased age confers a greater hazard while decreased z confers a greater hazard. To make HR comparisons of z with age, the reciprocal of the z HR is required.

**Table 5:** Survival probability statistical test summaries.

Model	Test	Chi-Square	DF	p-value
Accepted				
Dichotomous Age over Strata	Log-Rank	4.1784	1	0.0409
	Wilcoxon	3.4073	1	0.0649
Dichotomous Age and Gender over Strata	Log-Rank	12.7383	3	0.0052*
	Wilcoxon	13.5117	3	0.0043*
Hybrid				
Dichotomous z over Strata	Log-Rank	22.7597	1	< 0.0001
	Wilcoxon	14.9418	1	0.0001
Dichotomous z and Gender over Strata	Log-Rank	28.1863	3	< 0.0001*
	Wilcoxon	22.4886	3	< 0.0001*

The statistical tests findings for the various age an z related survival probability curves are provided with the degrees of freedom (DF). When comparing more than two survival curves (*), the hypothesis that all the curves were the same was tested against the alternative that at least one curve was different.

## Discussion

A technique for incorporating SL methods with epidemiologic analyses was illustrated. The approach used ensemble averaging based on bootstrap sampling. These preliminary findings indicate the hybrid approach provided benefits. With this data, the hybrid approach provided greater Az in the logistic regression modeling and greater hazard relationships in the survival analyses than that of the accepted approaches. The internal validity of our findings is supported by the analysis provided in the Appendix. This approach represents a framework that is easily generalized. We used the SL output as the input into LR model and survival analysis, essentially combining the strengths of the various modeling techniques. In this capacity, the SL device was operating as frontend preprocessing step for these accepted analysis techniques. Processing the SL output with these approaches provides a mechanism for converting the SL output into epidemiologic metrics (i.e. ORs and HRs). We used a relatively simple SL device to demonstrate the concept with a two-class problem. This specific approach can be extended to include more than two classes (e.g. death, greater than three, and five year survival benchmarks). The PNN applies to multiclass problems, as well, and multinomial logistic regression can address multiple level outcomes. It could be argued that the LR modeling was suboptimal because the time-to-event variable resolution was reduced to a coarser dichotomous variable. However for a specific set of variables, the LR output provides a different metric (i.e. probability of having a favorable outcome) than that provided by Cox regression (i.e. instantaneous relative risk). Thus, the resolution reduction is the price paid for an alternative output. More generally, the same hybrid approach is applicable for the output of any other type of SL method or decision device (e.g. support vector machines, kernel based partial least squares, or other types of neural networks).

There are several limitations with our findings. The analysis was performed with a limited number of samples derived retrospectively. Censoring limits the survival time estimations. Although the DE is a robust approach, there is no guarantee that it will converge indicating that the findings may be less than optimal. The generation termination limit, G = 3, was empirically set because we found that letting the process evolve over many generations produced weights that were too finely tuned and did not provide performance consistency between the training evaluation and the final score assessments. Because the dataset was limited, further evaluation using both simulation methods and holdout cross-validation with the z-score was provided in the Appendix. The findings from the hybrid modeling will require further evaluations with different datasets to show generalization. In principle to use a system as illustrated here in practice, the sampled patient population should be representative of stage-1 lung cancer patients in general. The operation of this system with new datasets would relegate this current dataset to assume the position of **w**_i _in the final score generation using prospective samples as **w**(without further training) to generate z for assessing survival probabilities or predicting favorable outcomes. The SL method was trained with a dichotomized survival output, which was the same output used to train the hybrid LR model. Therefore, the corresponding hybrid LR model (or the survival curve separation) could be confounded by the z variable if the choice of kernel or weights were suboptimal. Determining the optimal kernel was beyond the scope of this research.

Generalization of the LR model and incorporating kernel based techniques into epidemiologic survival analyses represents a diverse field of inquiry. Earlier research used a PNN and LR modeling to predict survival in early stage NSCLC but did not fuse the models [20]. Logistic regression is a member of a family of generalized linear models. Replacing the LR argument with various forms of smooth functions has provided benefits in the study of colon-cancer [21], heart-disease [22] and infant mortality [23]. Other researchers have incorporated univariate kernel density estimations for studying prostate-cancer [24], health disparities [25], and nutrient intake [26]. Similarly, univariate kernel density estimations have been used to estimate summary measures that were incorporated into LR modeling in fast-food consumption studies [27]. Our work differs from this other work in that the PNN application makes no assumption concerning the functional relationship of the variables under study and we incorporated the measures into LR.

## Conclusion

An SL methodology comprised of DE optimization, a kernel mapping, and stochastic ensemble averaging was presented as an illustration to generalize widely used analysis techniques. The technique gives the SL methodology an epidemiologic interpretation. Although we used a specific example, the framework applies to all situations where LR modeling and survival analysis are appropriate. The approach can be easily modified to include as many input variables as required and new samples can be added into the training procedure with the proper clinical feedback indicating the system can learn continually without computer processing demands due to its relative simplicity. The system will require further evaluation with different datasets before it can be applied in practice.

## Competing interests

The authors declare that they have no competing interests.

## Authors' contributions

MB is the primary author and was instrumental in designing and developing the work and performed data analyses. EEF is the secondary author, performed data analyses, assisted in developing the differential evolution and statistical learning methods with MB and JJH. TKO reviewed and edited the manuscript. WHL provided expertise used in the statistical learning developments, reviewed and edited the manuscript. WM was instrumental in the data collection protocol implementation, reviewed and edited the manuscript. ZC reviewed and edited the manuscript. FRK reviewed and edited the manuscript. SSR was the Principal Investigator for the protocol involving the tumor tissue and clinical data collection, reviewed and edited the manuscript. JJH is the senior author and played an important role in the development of the statistical learning system and data analyses. All authors have read and approved the final manuscript.

## Appendix

Additional evaluation was performed to assess the PNN z score method that included a simulation study and holdout cross-validation analysis.

### Simulation evaluation

A simulation was performed to assess the training, optimization, and patient scoring system shown in Figure 1 under ideal conditions. We used the same simulation methods with two correlated input features and non-linear separation boundary as described in our earlier work [4]. We used 200 samples per class giving 400 samples total as previously for the training dataset. We used this training dataset to estimate the sigma-weights using the algorithm described above (Figure 1). We used the same stochastic averaging (N _t _= 5, and N_sc _= 20) and bootstrap methods. We stopped the differential evolution optimization for G = 3 as above, which gave two sigma-weights (0.291156797, 0.0872920) with a training Az = 0.987. The training dataset was then used for **w**_i _in the score generation using independent data. We then simulated an evaluation dataset of the same dimension (200 per class giving 400 samples total) that was not used in the sigma-weight generation. These new samples were then used as **w **in the stochastic score generation and evaluated. This evaluation gave Az = 0.979. This shows in principle, the system is viable and that the training distribution must be representative of the population. It is also worth noting that the separation provided by this modified PNN system was larger than that described previously using a different statistical learning system when processing the same type of simulated datasets (i.e. Az ≈ 0.950).

### Holdout cross-validation

To assess the internal validity of the approach, we used the scheme shown in Figure 1 with one main difference. Two patient samples (one sample from each group) were selected at random and held out (i.e. leave two-out cross-validation) of the training process To slow the DE convergence, we set Cr = 0.1. The system comprised of the remaining n-2 patients was trained for 20 DE generations for each holdout pair. These n-2 samples were used for training and for generating training z scores (age combined with grade with the PNN) and Azs. For each DE generation, a bootstrap population was generated from the fixed n-2 population and an Az was generated. The weights that gave the largest Az for the 20 DE generations were used to generate the z scores for the two samples (holdout pair). We used stochastic averaging for the output scores, where 20 bootstrap populations were generated from the fixed n-2 training samples (generated 20 scores for each of the two left out samples). This process cycled (i.e. choosing another pair at random leaving a new n-2 training population for the next 20 DE generations) until all patients received a score. The resulting leave two out cross-validations gave Az = 0.700, indicating the approach was internally valid.
